# Chromosome Cohesion: A Cycle of Holding Together and Falling Apart

**DOI:** 10.1371/journal.pbio.0030094

**Published:** 2005-03-15

**Authors:** Jennifer Gerton

## Abstract

When a cell prepares to divide, the chromosomes need to separate at just the right moment. Regulating the cohesion of chromosomes is key to achieving this

All organisms have mechanisms to ensure that dividing cells produce new cells with the proper number of chromosomes. The dividing cell closely monitors that chromosomes are copied exactly once and then distributed correctly to daughter cells. After replication, the chromosomes (now comprising two chromatids) align at the center of the cell, and are attached to a structure known as the spindle apparatus. A key point of attachment is the centromere, a characteristic constriction carried by each chromosome. The spindle, which is composed of microtubules, pulls the chromatids apart so that two complete sets of chromosomes are gathered together at each pole of the cell, which can then divide. Cohesion between chromosome copies, which keeps the chromatids together until just the right time, therefore plays a critical part in this process.

Chromosome cohesion is established during S phase (when the chromosomes are replicated) and is then dissolved completely in metaphase to allow sister chromatids to come apart. The dissolution of cohesion is highly regulated; human cell lines that have defects in the regulation of cohesion show the hallmarks of cancer cells [1]. Furthermore, it has been suggested that the abnormal karyotypes that result in diseases such as Down syndrome are the result of the improper dissolution of chromosome cohesion [2]. Finally, mutation of a factor required to load cohesin—the protein complex responsible for chromosome cohesion—onto chromosomes appears to cause Cornelia de Lange syndrome, a clinically heterogeneous developmental disorder that may include facial dysmorphia, upper-extremity malformations, hirsutism, cardiac defects, growth and cognitive retardation, and gastrointestinal disorders [3,4,5].

Cohesion serves at least three roles in the cell with respect to accurate genome transmission. First, cohesion close to the centromere facilitates bi-orientation of chromosomes, such that each chromosome becomes attached to the two poles of the spindle [6]. Second, it prevents the splitting of chromosomes until all bipolar attachments are made [6]. The function of cohesion at the centromere is presumably to oppose the force of microtubules, which pull the chromosomes to opposite spindle poles; this force is not exerted along the chromosome arms, which means that cohesion at centromeres and along arms is functionally distinct. Third, cohesion along chromosome arms may be essential for proper chromosome condensation [7,8], although the function of cohesion at chromosome arms is something of a mystery.

## Differences between Arms and Centromeres

Cohesion in eukaryotic cells is mediated by a multi-subunit protein complex called cohesin. Cohesin consists of four proteins: Smc1, Smc3, Scc1/Mcd1 (also known as kleisin), and Scc3 (SA2). The Smc (structural maintenance of chromosomes) proteins form intramolecular coiled coils that have been observed in the electron microscope to form a V shape with sides that are 50 nm long [9]. The cohesin complex has been proposed to form a ring structure that encircles sister chromatids [10]. Alternately, two rings may snap sisters together via interactions between the coiled coils of the Smc proteins [11]. All members of the cohesin complex are essential in budding yeast, Saccharomyces cerevisiae, since mutation results in the precocious dissociation of sister chromatids. Functional orthologs of these proteins exist in all eukaryotes.

**Figure 1:**
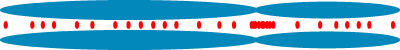
Cohesin Sites for Sister Chromatids of Chromosome I in S. cerevisiae Cohesin sites (red ovals) are concentrated at the centromere/pericentric region (where the two chromatids are “pinched”), but also occur along the arms of the chromatids.

There are at least two types of cohesin sites: (1) cohesin associated with the centromere and the nearby pericentric domain, and (2) cohesin associated with chromosome arms [12,13,14,15]. In S. cerevisiae, cohesin at centromeric and pericentric domains is spread over a broad region (up to 50 kb), large quantities of the protein complex are bound, and binding is not affected by the natural transcriptional and coding status of the regions that are occupied. By contrast, binding sites in arms tend to be much smaller (about 1 kb)—at least in S. cerevisiae, where they have been most extensively characterized—and of lower intensity, and are spaced at approximately every 11 kb (see Figure 1). Cohesin in arms localizes to regions lacking transcription in yeast [12,16,17]. This reinforces the view that there may be functional differences in arm and pericentric cohesion and perhaps different mechanisms to load cohesin, as has been proposed for cohesin on meiotic chromosomes for S. pombe [18]. A unifying feature of all cohesin-binding sites in S. cerevisiae is high AT (adenine and thymine) content [12,15].

Another important difference between cohesin binding along arms and at centromeres is that the arm sites do not appear to be dependent on a DNA consensus sequence, whereas binding to pericentric regions requires specific centromere sequence [13,14]. The S. cerevisiae centromere sequence is composed of three DNA elements (CDEI, CDEII, and CDEIII). Studies of cohesion at the centromere reveal that as little as 100 bp (a portion of CDEII and the entire CDEIII) are required to direct cohesion [13,14,19]. Mutations in the protein Ndc10 have also been shown to affect cohesin deposition at centromeres. Ndc10 forms part of a structure known as the kinetochore, which forms around the centromere and is responsible for the attachment to the spindle; establishment and maintenance of cohesion at pericentric regions may therefore rely on both the centromere sequence and kinetochore function [13,20]. Presumably both arm and pericentric cohesion are important for chromosome dynamics, but the functional differences between the two are not well understood.

## Cohesion—It's Just a Phase

**Figure 2:**
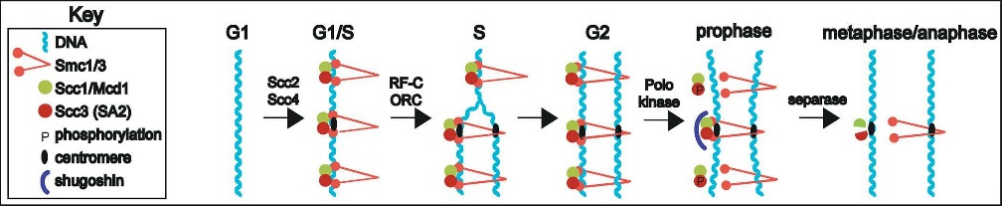
Behavior of Cohesin during the Cell Cycle One cohesin complex is depicted at each site for the sake of simplicity; at the centromere especially there are likely to be many complexes. Cohesion is represented as occurring via the “encircling” model; other models have been proposed.

Cohesion can be divided into four phases that occur during the cell cycle (Figure 2): (1) deposition in G1 (the gap in the cell cycle before S phase), (2) establishment in S phase, (3) maintenance in G2 (the gap between S and mitosis), and (4) dissolution in mitosis. During G1, Scc2 and Scc4 are responsible for loading cohesin onto unreplicated double-stranded DNA [21]. Then, during S phase, several proteins are involved in establishment of cohesion between replicated chromatids. Eco1 and Chl1 are required for establishing cohesion but not for maintenance [22,23,24]. The associations between cohesion and DNA replication have led to a model whereby cohesion is established coincident with the passage of the replication fork [25]. This requires an alternative replication factor C (RF-C) complex [26,27,28] and may require the origin recognition complex (ORC) [29]. Cohesion is maintained during G2 by the cohesin complex, and is eventually dissolved in mitosis to allow sister chromatids to separate.

The dissolution of cohesion is regulated by at least two mechanisms. First, subunits of the complex may be phosphorylated, which facilitates their removal. In S. cerevisiae and human cells, phosphorylation of Scc1/Mcd1 by Polo kinase makes it a better substrate for proteolysis [30,31,32]. In this issue of *PLoS Biology*, one of two related papers exploring the regulation of cohesin in vertebrates shows that phosphorylation of Scc3 (SA2) by Polo-like kinase is apparently sufficient to allow dissociation from chromosome arms, which occurs during prophase [32]. In Xenopus extracts, phosphorylation of cohesin also depends on Polo-like kinase, and this phosphorylation reduces the ability of cohesin to bind to chromatin [8].

The second mechanism that can facilitate the dissolution of cohesin is proteolysis; this may be particularly important at centromeres. The Scc1/Mcd1 component of the cohesin complex is cleaved by a separase (Esp1) whose activity is held in check by a securin (Pds1) until separation at the metaphase-to-anaphase transition [33,34]. Separase is a cysteine protease that cleaves Scc1/Mcd1, presumably resulting in the cohesin complex falling apart and being unable to hold sister chromatids together.

Scc1/Mcd1 at pericentric regions is protected from phosphorylation during prophase—and therefore dissociation from chromosomes is prevented—by proteins known as shugoshins [35,36,37]. In the second paper on cohesin in this issue of *PLoS Biology*, McGuinness et al. show that shugoshin specifically protects Scc3 (SA2) at the centromere, so that centromeric cohesion is preserved until the chromosomes are ready to separate [35]. Vertebrate shugoshin has been shown to have a strong microtubule-binding domain [36] and is found at the kinetochore [37]. Recent evidence suggests that shugoshin in S. cerevisiae may sense tension between sister chromatids, acting as part of a spindle checkpoint that monitors whether chromosomes are properly aligned on the mitotic spindle [38]. It is currently unclear why the cell has two mechanisms to dissociate cohesin from chromosomes, although it is interesting to speculate that this could be related to different functions of cohesin at chromosome arms versus pericentric domains. For instance, cohesin in chromosome arms may help to organize or condense chromosomes, whereas cohesin at centromeres may be more directly involved in chromosome bi-orientation at the spindle and segregation. These functions may be important during different phases of the cell cycle.

## A Link between Chromatin and Cohesin

Several results suggest that transcription and cohesin binding are incompatible. In Drosophila, one of the components that loads cohesin (Nipped-B or Scc2) has also been shown to be required for long-range promoter–enhancer interactions [39,40]. One model proposed to explain this result is that cohesin can prevent long-range promoter–enhancer interactions and that removal of cohesin can restore these interactions and allow transcription to occur [41]. In this model, Nipped-B or Scc2 can act as both a loading factor and an unloading factor for cohesin. This model further speculates that rather than Cornelia de Lange syndrome stemming from a cohesin loading defect, the failure to unload cohesin from regions that need to be transcribed leads to transcriptional defects that cause the syndrome. In S. cerevisiae it has been shown that driving transcription through a centromere via an inducible promoter prevents cohesin from associating and results in chromosome missegregation and cell death [13]. Cohesin is found at the boundaries of the HMR locus, the right telomere of Chromosome III, and the RDN1 array, all regions of silent chromatin [16]. Cohesin localizes to intergenic regions where transcription is converging [12,17].

Since transcription and chromatin configuration are intimately related, it is possible that chromatin may play an important role in the localization of cohesin. Indeed, the chromatin remodeling complex RSC (remodels the structure of chromatin) has been shown to be important for establishment of cohesin binding [42], and another study suggests RSC is particularly important for cohesin association with chromosome arms [43]. The chromatin remodeling complex ISWI (SNF2h) has been shown to be essential for cohesin to localize to Alu repeats (certain DNA sequences) in human cells [44]. The possibility also exists that cohesin itself may influence transcriptional status and act as a transcriptional boundary [39,40,45]. The preferential location of cohesin in heterochromatin in pericentric regions in S. pombe also supports the idea that chromatin modification/structure is a key determinant of cohesin localization [46,47]. It is interesting to speculate that chromatin differences and transcriptional differences between chromosome arms and centric regions will turn out to be related to different mechanisms for loading and removal of cohesin from these regions.

While one of the primary roles for chromosome cohesion in bi-orientation and mitotic chromosome segregation is well-established, the complexities of the regulation of cohesion are still being discovered. Cohesin may be involved in multiple ways in chromosome dynamics. Future studies focusing on the differences between cohesion at chromosome arms versus pericentric domains and the link between cohesion and transcription will likely yield very interesting insights into the function of the cohesin complex in the maintenance of genome integrity.

## References

[pbio-0030094-b1] Jallepalli PV, Waizenegger IC, Bunz F, Langer S, Speicher MR (2001). Securin is required for chromosomal stability in human cells. Cell.

[pbio-0030094-b2] Shonn MA, McCarroll R, Murray AW (2002). Spo13 protects meiotic cohesin at centromeres in meiosis I. Genes Dev.

[pbio-0030094-b3] Krantz ID, McCallum J, DeScipio C, Kaur M, Gillis LA (2004). Cornelia de Lange syndrome is caused by mutations in NIPBL, the human homolog of Drosophila melanogaster Nipped-B. Nat Genet.

[pbio-0030094-b4] Gillis LA, McCallum J, Kaur M, DeScipio C, Yaeger D (2004). NIPBL mutational analysis in 120 individuals with Cornelia de Lange syndrome and evaluation of genotype–phenotype correlations. Am J Hum Genet.

[pbio-0030094-b5] Tonkin ET, Wang TJ, Lisgo S, Bamshad MJ, Strachan T (2004). NIPBL, encoding a homolog of fungal Scc2-type sister chromatid cohesion proteins and fly Nipped-B, is mutated in Cornelia de Lange syndrome. Nat Genet.

[pbio-0030094-b6] Tanaka T, Fuchs J, Loidl J, Nasmyth K (2000). Cohesin ensures bipolar attachment of microtubules to sister centromeres and resists their precocious separation. Nat Cell Biol.

[pbio-0030094-b7] Guacci V, Koshland D, Strunnikov A (1997). A direct link between sister chromatid cohesion and chromosome condensation revealed through the analysis of MCD1 in S. cerevisiae. Cell.

[pbio-0030094-b8] Sumara I, Vorlaufer E, Stukenberg PT, Kelm O, Redemann N (2002). The dissociation of cohesin from chromosomes in prophase is regulated by Polo-like kinase. Mol Cell.

[pbio-0030094-b9] Haering CH, Lowe J, Hochwagen A, Nasmyth K (2002). Molecular architecture of SMC proteins and the yeast cohesin complex. Mol Cell.

[pbio-0030094-b10] Gruber S, Haering CH, Nasmyth K (2003). Chromosomal cohesin forms a ring. Cell.

[pbio-0030094-b11] Milutinovich M, Koshland DE (2003). Molecular biology. SMC complexes—Wrapped up in controversy. Science.

[pbio-0030094-b12] Glynn EF, Megee PC, Yu HG, Mistrot C, Unal E (2004). Genome-wide mapping of the cohesin complex in the yeast Saccharomyces cerevisiae. PLoS Biol.

[pbio-0030094-b13] Tanaka T, Cosma MP, Wirth K, Nasmyth K (1999). Identification of cohesin association sites at centromeres and along chromosome arms. Cell.

[pbio-0030094-b14] Megee PC, Mistrot C, Guacci V, Koshland D (1999). The centromeric sister chromatid cohesion site directs Mcd1p binding to adjacent sequences. Mol Cell.

[pbio-0030094-b15] Blat Y, Kleckner N (1999). Cohesins bind to preferential sites along yeast chromosome III, with differential regulation along arms versus the centric region. Cell.

[pbio-0030094-b16] Laloraya S, Guacci V, Koshland D (2000). Chromosomal addresses of the cohesin component Mcd1p. J Cell Biol.

[pbio-0030094-b17] Lengronne A, Katou Y, Mori S, Yokobayashi S, Kelly GP (2004). Cohesin relocation from sites of chromosomal loading to places of convergent transcription. Nature.

[pbio-0030094-b18] Kitajima TS, Yokobayashi S, Yamamoto M, Watanabe Y (2003). Distinct cohesin complexes organize meiotic chromosome domains. Science.

[pbio-0030094-b19] Megee PC, Koshland D (1999). A functional assay for centromere-associated sister chromatid cohesion. Science.

[pbio-0030094-b20] Weber SA, Gerton JL, Polancic JE, DeRisi JL, Koshland D (2004). The kinetochore is an enhancer of pericentric cohesin binding. PLoS Biol.

[pbio-0030094-b21] Ciosk R, Shirayama M, Shevchenko A, Tanaka T, Toth A (2000). Cohesin's binding to chromosomes depends on a separate complex consisting of Scc2 and Scc4 proteins. Mol Cell.

[pbio-0030094-b22] Toth A, Ciosk R, Uhlmann F, Galova M, Schleiffer A (1999). Yeast cohesin complex requires a conserved protein, Eco1p(Ctf7), to establish cohesion between sister chromatids during DNA replication. Genes Dev.

[pbio-0030094-b23] Skibbens RV, Corson LB, Koshland D, Hieter P (1999). Ctf7p is essential for sister chromatid cohesion and links mitotic chromosome structure to the DNA replication machinery. Genes Dev.

[pbio-0030094-b24] Skibbens RV (2004). Chl1p, a DNA helicase-like protein in budding yeast, functions in sister-chromatid cohesion. Genetics.

[pbio-0030094-b25] Uhlmann F, Nasmyth K (1998). Cohesion between sister chromatids must be established during DNA replication. Curr Biol.

[pbio-0030094-b26] Mayer ML, Gygi SP, Aebersold R, Hieter P (2001). Identification of RFC(Ctf18p, Ctf8p, Dcc1p): An alternative RFC complex required for sister chromatid cohesion in S. cerevisiae. Mol Cell.

[pbio-0030094-b27] Hanna JS, Kroll ES, Lundblad V, Spencer FA (2001). Saccharomyces cerevisiae CTF18 and CTF4 are required for sister chromatid cohesion. Mol Cell Biol.

[pbio-0030094-b28] Warren CD, Eckley DM, Lee MS, Hanna JS, Hughes A (2004). S-phase checkpoint genes safeguard high-fidelity sister chromatid cohesion. Mol Biol Cell.

[pbio-0030094-b29] Suter B, Tong A, Chang M, Yu L, Brown GW (2004). The origin recognition complex links replication, sister chromatid cohesion and transcriptional silencing in Saccharomyces cerevisiae. Genetics.

[pbio-0030094-b30] Alexandru G, Uhlmann F, Mechtler K, Poupart MA, Nasmyth K (2001). Phosphorylation of the cohesin subunit Scc1 by Polo/Cdc5 kinase regulates sister chromatid separation in yeast. Cell.

[pbio-0030094-b31] Hornig NC, Uhlmann F (2004). Preferential cleavage of chromatin-bound cohesin after targeted phosphorylation by Polo-like kinase. EMBO J.

[pbio-0030094-b32] Hauf S, Roitinger E, Koch B, Dittrich C, Mechtler K (2005). Dissociation of cohesin from chromosome arms and loss of arm cohesion during early mitosis depends on phosphorylation of SA2. PLoS Biol.

[pbio-0030094-b33] Ciosk R, Zachariae W, Michaelis C, Shevchenko A, Mann M (1998). An ESP1/PDS1 complex regulates loss of sister chromatid cohesion at the metaphase to anaphase transition in yeast. Cell.

[pbio-0030094-b34] Uhlmann F, Lottspeich F, Nasmyth K (1999). Sister-chromatid separation at anaphase onset is promoted by cleavage of the cohesin subunit Scc1. Nature.

[pbio-0030094-b35] McGuinness BE, Hirota T, Kudo NR, Peters JM, Nasmyth K (2005). Shugoshin prevents dissociation of cohesin from centromeres during mitosis in vertebrate cells. PLoS Biol.

[pbio-0030094-b36] Salic A, Waters JC, Mitchison TJ (2004). Vertebrate shugoshin links sister centromere cohesion and kinetochore microtubule stability in mitosis. Cell.

[pbio-0030094-b37] Kitajima TS, Kawashima SA, Watanabe Y (2004). The conserved kinetochore protein shugoshin protects centromeric cohesion during meiosis. Nature.

[pbio-0030094-b38] Indjeian VB, Stern BM, Murray AW (2005). The centromeric protein Sgo1 is required to sense lack of tension on mitotic chromosomes. Science.

[pbio-0030094-b39] Rollins RA, Korom M, Aulner N, Martens A, Dorsett D (2004). Drosophila nipped-B protein supports sister chromatid cohesion and opposes the stromalin/Scc3 cohesion factor to facilitate long-range activation of the cut gene. Mol Cell Biol.

[pbio-0030094-b40] Rollins RA, Morcillo P, Dorsett D (1999). Nipped-B, a Drosophila homologue of chromosomal adherins, participates in activation by remote enhancers in the cut and Ultrabithorax genes. Genetics.

[pbio-0030094-b41] Dorsett D (2004). Adherin: Key to the cohesin ring and Cornelia de Lange syndrome. Curr Biol.

[pbio-0030094-b42] Baetz KK, Krogan NJ, Emili A, Greenblatt J, Hieter P (2004). The ctf13-30/CTF13 genomic haploinsufficiency modifier screen identifies the yeast chromatin remodeling complex RSC, which is required for the establishment of sister chromatid cohesion. Mol Cell Biol.

[pbio-0030094-b43] Huang J, Hsu JM, Laurent BC (2004). The RSC nucleosome-remodeling complex is required for cohesin's association with chromosome arms. Mol Cell.

[pbio-0030094-b44] Hakimi MA, Bochar DA, Schmiesing JA, Dong Y, Barak OG (2002). A chromatin remodelling complex that loads cohesin onto human chromosomes. Nature.

[pbio-0030094-b45] Hagstrom KA, Meyer BJ (2003). Condensin and cohesin: More than chromosome compactor and glue. Nat Rev Genet.

[pbio-0030094-b46] Bernard P, Maure JF, Partridge JF, Genier S, Javerzat JP (2001). Requirement of heterochromatin for cohesion at centromeres. Science.

[pbio-0030094-b47] Nonaka N, Kitajima T, Yokobayashi S, Xiao G, Yamamoto M (2002). Recruitment of cohesin to heterochromatic regions by Swi6/HP1 in fission yeast. Nat Cell Biol.

